# DESIGNING A STATE-BASED SOCIAL INSURANCE PROGRAM FOR LONG-TERM SERVICES AND SUPPORTS

**DOI:** 10.1093/geroni/igz038.2123

**Published:** 2019-11-08

**Authors:** Marc A Cohen, Benjamin Veghte, Eileen J Tell, Alexandra L Bradley

**Affiliations:** 1 University of Massachusetts Boston Gerontology Institute, LeadingAge LTSS Center @UMass Boston, Boston, Massachusetts, United States; 2 National Academy of Social Insurance, Washington, District of Columbia, United States; 3 ET Consulting, Belmont, Massachusetts, United States

## Abstract

The fundamental LTSS financing problem today is the absence of an effective insurance mechanism. To achieve universal coverage, social insurance is required. This report identifies key design questions for states to consider in crafting an LTSS social insurance program, outlines a range of vetted approaches states could adopt, and describes the building blocks and tradeoffs associated with these options. This analysis was developed during a year of deliberations by a Study Panel of 15 experts in LTSS with a variety of perspectives. States must answer two critical first-order questions. First, who is the program seeking to help – only the elderly, or all people with disabilities? Only those who start paying into the program now, or current retirees as well? Second, how will the program be financed – through a payroll tax, an income tax, or some other dedicated revenue source? Additional considerations follow from these two overarching questions.

